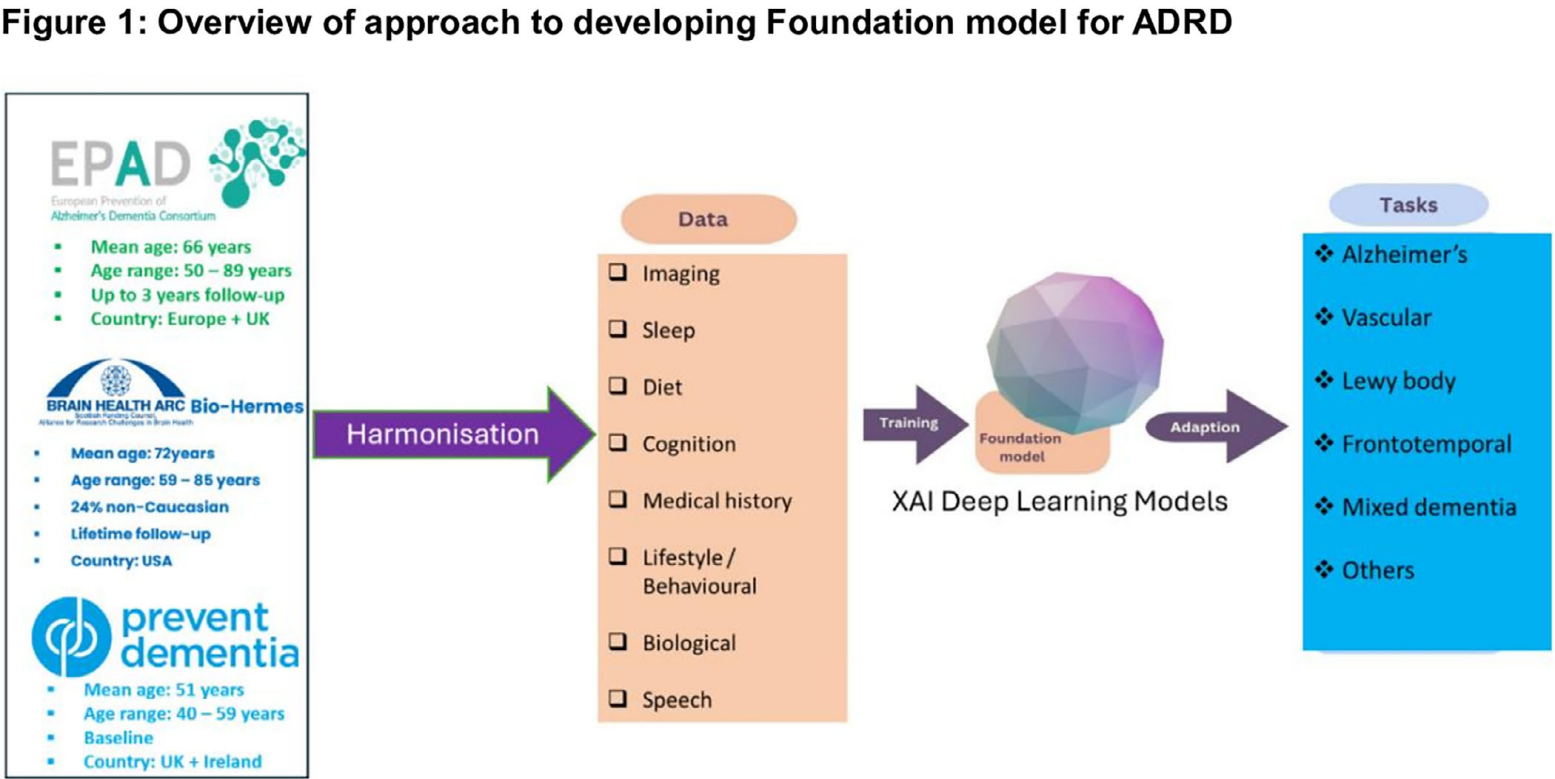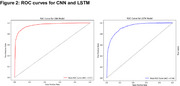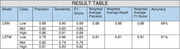# Developing Foundation Model for Early Detection of Alzheimer's Disease and Related Dementias (ADRD) from midlife

**DOI:** 10.1002/alz70860_106904

**Published:** 2025-12-23

**Authors:** Samuel O. Danso, Ibrahim Alqatawneh, Adewale Samuel Owo, Tamlyn J Watermeyer, Joe Butler, Poorna Gunasekera

**Affiliations:** ^1^ School of Computer Science and Engineering, University of Sunderland, Sunderland, England, United Kingdom; ^2^ University of Edinburgh, Edinburgh, United Kingdom; ^3^ University of Sunderland, Sunderland, England, United Kingdom; ^4^ University of Sunderland, Sunderland, United Kingdom; ^5^ Edinburgh Dementia Prevention, Centre for Clinical Brain Sciences, College of Medicine and Veterinary Medicine, University of Edinburgh, Edinburgh, United Kingdom; ^6^ University of Northumbria, Newcastle upon tyne, England, United Kingdom; ^7^ School of Psychology, University of Sunderland, Sunderland, United Kingdom; ^8^ National Institute of Health Applied Research Collaboration North East & Cumbria, Newcastle‐Upon‐Tyne, England, United Kingdom; ^9^ Unviversity of Plymouth, Plymouth, England, United Kingdom

## Abstract

**Background:**

While recent development of Artificial Intelligence (AI)‐based approaches have demonstrated to be effective in predicting risk of ADRD, these have mostly focused on AD subtype, aged and homogenous populations (Grueso et al, 2022; Rahim et al., 2023), thereby limiting their applicability to other types of ADRD and younger populations. Inspired by earlier work (Danso et al 2021), we propose an AI‐based deep‐learning framework for early detection of ADRD based on heterogeneous and diverse population from midlife (Figure 1).

**Method:**

We obtained two datasets from the European Prevention of Alzheimer's Dementia‐ EPAD (*n* = 2096) and PREVENT Dementia Programme (*n* = 700) available online (AD workbench, 2020). Following procedures described in Danso et al (2018) a harmonised cohort was curated containing individuals with no diagnosis of dementia. We then created three risk groups (High risk = ApoE4 allele and family history of AD; Medium risk = ApoE4 allele but no family history of AD; Low risk = no ApoE4 allele and no family history of AD) following the risk definition by Ritchie & Ritchie (2012). Convolutional Neural Network (CNN) and Long‐ Short Term Memory (LSTM) models were developed using 5‐fold cross validation and then applied optimisation procedures to obtain optimal parameters for the trained models.

**Result:**

The harmonisation resulted in a cohort (*n* = 2796; mean age =62; range = 40 – 89years; female =57.5%, Caucasian = 95%), containing medical history, physiological, lifestyle, neuroimaging, and sociodemographic features. Overall, CNN outperformed LSTM by 7% points for accuracy and f1‐score (Table 1), with mean AUROC scores of 97% and 94% respectively (Figure 2), and mean validation loss scores (CNN = 0.36; LSTM =0.46).

**Conclusion:**

The superior performance of CNN is consistent with the literature and the relatively low validation loss demonstrates its generalisability. While this model is currently optimised for AD with limited features, a Transfer Learning paradigm is being employed to further train the CNN model to predict risk of other AD sub‐types after including BioHermes dataset into pipeline. Future work will also explore modifications of the CNN architecture for multimodal features with explainability capabilities.